# Outcomes after toxic alcohol poisoning: a systematic review protocol

**DOI:** 10.1186/s13643-018-0926-z

**Published:** 2018-12-28

**Authors:** Carol Wang, Daniel Samaha, Swapnil Hiremath, Lindsey Sikora, Manish M. Sood, Salmaan Kanji, Edward G. Clark

**Affiliations:** 1 Department of Medicine, University of Ottawa, 162 Parkdale Avenue, Ottawa, Ontario K1Y 1J8 Canada; 2 The Ottawa Hospital – Riverside Campus, 1967 Riverside Drive, Ottawa, Ontario K1H 7W9 Canada; 3 Division of Nephrology, Department of Medicine, University of Ottawa, 1967 Riverside Drive, Ottawa, K1H 7W9 Ontario Canada; 4 The Ottawa Hospital Research Institute, University of Ottawa, 501 Smyth Road, Ottawa, Ontario K1H 8L6 Canada; 5 Health Sciences Library, University of Ottawa, 451 Smyth Road, Ottawa, K1H 8M5 Ontario Canada

**Keywords:** Toxic alcohol, Intoxication, Poisoning, Extracorporeal treatment, Dialysis, Antidotes, Systematic review

## Abstract

**Background:**

Toxic alcohols have been implicated in accidental ingestions and intentional exposures. Recognition of toxic alcohol poisoning is challenging. The main treatment modalities include antidotes with alcohol dehydrogenase inhibitors and dialysis. Current guidelines exist for both methanol and ethylene glycol intoxication. However, treatment consensus related to other toxic alcohols is limited. Furthermore, uncertainties regarding thresholds for when to initiate antidotes and dialysis persist. As a consequence, variations exist in the interventions utilized for management of all toxic alcohol poisonings. To our knowledge, no prior systematic review of clinical outcomes of toxic alcohols exists. The objective of this study is to summarize existing evidence on short- and long-term outcomes of patients following toxic alcohol poisonings, including methanol, ethylene glycol, isopropanol, propylene glycol, and diethylene glycol.

**Methods:**

A literature search in PubMed, MEDLINE, and EMBASE will be performed based on pre-determined criteria. There will be no restrictions on publication dates or languages. The search will be supplemented by manual scan of bibliographies of eligible studies and gray literature assessment. Observational studies and clinical trials will be included in this review. Once eligible studies have been selected based on pre-specified criteria, two investigators will extract relevant data independently and perform quality assessment per validated tools. A pooled analysis of mortality and short- and long-term secondary outcomes will be performed. Pre-specified subgroup analyses will be performed according to the type of toxic alcohol intoxication, mode of renal replacement therapy, and medical interventions received. A meta-analysis will be performed if three or more studies with similar populations, type of toxic alcohol poisoning, and outcome measures, as well as adequate quality, are identified. This review will be reported according to the recommendations of the Preferred Reporting Items for Systematic Review and Meta-Analyses (PRISMA) Statement.

**Discussion:**

This systematic review aims to synthesize current evidence in the short- and long-term outcomes of post-toxic alcohol poisoning. The results will enhance the understanding of patient morbidity and mortality after toxic alcohol poisoning, help inform uniform concrete management guideline development, identify gaps in the current state of knowledge, and provide evidence to help implement post-treatment follow-up.

**Systematic review registration:**

PROSPERO CRD42018101955

**Electronic supplementary material:**

The online version of this article (10.1186/s13643-018-0926-z) contains supplementary material, which is available to authorized users.

## Background

Toxic alcohols include methanol, ethylene glycol, isopropyl alcohol, diethylene glycol, and propylene glycol [1, 2]. These alcohols have been implicated in accidental ingestions as well as intentional exposures in suicides and homicides [1]. They are readily obtainable through automotive and hardware stores as well as household detergents [1]. Between 2005 and 2014, methanol made up 5.7% while ethylene glycol constituted 5.9% of all intoxications managed with extracorporeal renal replacement therapy (ECRT) in Canada [3]. In the USA, approximately 50% of ECRTs performed over the same time period were for ethylene glycol, lithium, and salicylates intoxications [3]. Methanol poisoning was the most common indication for the management with renal replacement therapy amongst toxic alcohol intoxications, others of which include ethylene glycol, isopropanol, propylene glycol, and diethylene glycol [3].

Recognition of toxic alcohol poisoning is challenging due to non-specific clinical presentation and that patients are often unable to provide a history of exposure at presentation [1, 2]. Clinical manifestations may evolve with the formation of toxic metabolites and are further compounded by co-ingestions [1]. Another element of challenge for diagnosis is a negative parent compound level in the serum if there is a delay in clinical presentation and the initial compound has been metabolized [1]. Prompt identification and initiation of therapy have important prognostic implications [4].

The toxicity of methanol and ethylene glycol arises from their respective metabolites while the parent compound is relatively innocuous [4]. The parent compounds of methanol and ethylene glycol are metabolized by alcohol dehydrogenase [1]. Formic acid is the breakdown product of methanol, which has the propensity to accumulate in the retina and basal ganglia to produce visual disturbances and blindness [1, 2]. Ethylene glycol is eventually converted to oxalic acid, which causes renal and cranial nerve damage secondary to calcium oxalate deposition [1, 2, 5]. Metabolic acidosis and inebriation are common features of methanol and ethylene glycol metabolite toxicity [4]. The American Academy of Clinical Toxicology has outlined recommendations for antidote therapy with either ethanol or fomepizole as well as indications for hemodialysis [5–7]. Hemodialysis effectively removes both the parent compound and its toxic metabolites in addition to correcting metabolic disturbances [5, 6, 8].

In contrast to methanol and ethylene glycol, the toxicity of isopropanol arises from the parent compound [9]. Isopropanol is commonly found in rubbing alcohol and hand sanitizers and utilized as solvent or coating for industrial purposes [9]. Non-specific presentations associated with isopropanol poisoning include altered level of consciousness, respiratory depression, gastritis, acute pancreatitis, hypotension, and lactic acidosis [1, 2, 9]. Alcohol dehydrogenase inhibitors are contraindicated due to the associated accumulation of parent compound while its metabolite acetone is non-toxic [2, 9]. Isopropanol is excreted by the kidneys and toxicity is mainly managed supportively, but hemodialysis is approximately 52 times more effective for the removal of isopropanol [1, 9].

Propylene glycol and diethylene glycol toxicity are less commonly described in the literature. Propylene glycol is a common solvent for parenteral medications such as lorazepam, hence associated with high-dose infusion [10]. The compounds and their metabolites may precipitate metabolic acidosis and acute kidney injury [10–12]. Dialysis has been the main treatment modality for both toxicities while alcohol dehydrogenase inhibitor administration has been described in the literature for the management of diethylene glycol poisoning [10–13]. In some cases, hemodialysis has been initiated for the management of severe acidosis in the context of multiorgan failure due to propylene glycol and diethylene glycol intoxication for which treatment guidelines are not currently available [1].

Recommendations for the management of methanol poisoning have been put forth by the Extracorporeal Treatments in Poisoning (EXTRIP) workgroup and American Academy of Clinical Toxicology in the form of published guidelines for both methanol and ethylene glycol intoxications [5–7]. Unfortunately, it appears that the therapeutic benefits of hemodialysis are limited in cases where the toxic alcohols and metabolites have been metabolized and clinical sequelae are established [8]. Furthermore, consensus on thresholds for when to initiate alcohol dehydrogenase inhibitor and dialysis has yet been established [1, 2, 4]. Similar treatment guidelines and established standard of care have not been formulated for other types of toxic alcohols. Despite the presence of treatment guidelines for methanol and ethylene glycol poisoning, variations exist in their intervention strategies. For instance, the utilization of antidote monotherapy versus combination with renal replacement therapy, as well as the addition of adjunctive therapies such as folate in methanol poisoning and pyridoxine in ethylene glycol intoxication, is not standardized [1, 4]. Ethylene glycol and methanol concentration thresholds at which fomepizole can be safely discontinued remain to be defined [4]. The vast majority of existing literature on toxic alcohol poisoning is in the form of observational studies and systematic reviews on its epidemiology, clinical presentations, and management. Although predictors for negative long-term consequences of toxic alcohol poisoning have been described [14], there is an overall paucity of data focusing on patient outcomes. Short-term patient outcomes have been mentioned as part of the reports focusing on clinical presentations and management of toxic alcohol poisoning instead of being the focus of studies. Individual observational studies with small sample sizes have examined the neurological and renal sequelae of methanol and diethylene glycol poisoning with variable lengths of follow-up ranging from 2 to 18 months [15–17]. To our knowledge, no prior systematic review of short- and long-term outcomes of toxic alcohols has been published. Toxic alcohol poisonings such as methanol intoxication continue to result in high morbidity and mortality likely due to the delayed presentation to medical care and challenges in establishing the correct diagnosis [18]. This article describes the protocol for a systematic review aimed at summarizing existing evidence on short- and long-term outcomes of adult patients following toxic alcohol poisoning.

## Methods

The objective of this study is to summarize the existing evidence regarding short-term and long-term clinical outcomes of patients after toxic alcohol poisoning. The systematic review protocol is reported in accordance with the PRISMA-P 2015 Checklist (see Additional file 1). This protocol was registered with the International Prospective Register of Systematic Reviews (PROSPERO) CRD42018101955. A public trial of amendments to this systematic review protocol will be reflected in PROSPERO.

### Eligibility criteria

All studies that have reported on short-term and/or long-term outcomes of adult patients > 18 years old who presented with toxic alcohol poisoning are eligible. Both interventional and observational trials will be included. Case reports and case series consisting of less than or equal to five cases will be excluded. No restrictions will be placed on the study duration, study period, or date of publication. Furthermore, the pre-specified criteria detailed below must also be met.

#### Types of participants

We will include studies with patients 18 years of age or older diagnosed with toxic alcohol poisoning (specifically methanol, ethylene glycol, isopropanol, propylene glycol, and diethylene glycol poisoning). There are four groups of interest: (1) those treated with dialysis (this refers to extracorporeal interventions such as hemodialysis, continuous hemofiltration, hemoperfusion, continuous renal replacement therapy, and peritoneal dialysis, in this context), (2) those treated with specific antidote therapies fomepizole and/or ethanol, (3) those treated with both dialysis and specific antidote therapy, and (4) those treated with other medical treatments or supportive therapy only.

#### Exposure/intervention

The diagnosis of toxic alcohol poisoning may be made according to the presence of toxic alcohol(s) measured in the blood. Other criteria for toxic alcohol poisoning will also be included if they are clearly defined.

#### Outcomes

Clinical outcomes of interest are separated into short- and long-term, whereby short-term outcomes encompass clinically relevant end points (defined below) between the time of intoxication and hospital discharge. Long-term outcomes span up to 10 years post-hospitalization The primary outcome of interest in this systematic review is mortality at any time point reported. Short-term secondary renal outcomes include whether dialysis was undertaken for acute kidney injury due to toxic alcohol ingestion, type of dialysis, renal recovery at the time of hospital discharge, and duration of dialysis if renal recovery was achieved at discharge. Long-term secondary renal outcomes refer to ongoing dialysis dependence after hospital discharge, renal recovery post-discharge, duration of dialysis, and progression to end-stage renal disease requiring transplantation. Of note, renal recovery is defined as independence from renal replacement therapy [19, 20]. Short-term toxin-associated complications include dependence on cardiopulmonary supports (such as assisted ventilation and vasopressors), altered level of consciousness, and duration of hospitalization. Both the short- and long-term toxin-mediated complications such as vision loss due to methanol, neurologic deficits, and cardiovascular events will also be recorded.

### Search strategy

PubMed, MEDLINE (via Ovid), and EMBASE (via Ovid) were searched initially for prior systematic reviews that have addressed similar topics. PROSPERO was also searched to ensure a similar systematic review study protocols has not been registered. No prior studies of our topic of interest have been identified. The following databases will be searched by a health sciences librarian (LS) during the electronic component of the systematic review: MEDLINE and MEDLINE in Process (via OVID), EMBASE Classic + EMBASE (via OVID), Cochrane’s Central Registry for Randomized Controlled Trials, CENTRAL (via OVID), and PubMed. A search strategy will be developed in MEDLINE and then translated into the other databases, as appropriate (see Appendix). All databases will be searched from the date of inception to July 3, 2018. There are no language exclusion criteria nor any other publication restrictions. All references will be entered into the citation manager, Endnote (version X9, Clarivate Analytics Inc., Philadelphia, PA), for processing. Supplemental searching will include a manual scan of the bibliographies of eligible studies, as well as gray literature searching in clinical trial registries such as clinicaltrials.gov and Google Scholar. The first three pages of the Google Scholar search will be screened for relevant titles. Studies will be screened by two reviewers (CW and EC) in the systematic review software, Covidence (Veritas Health Innovation LTD). Screening will occur at two levels: title/abstract and then full-text screening.

### Study records

#### Data management

Relevant data will be extracted from Covidence (Veritas Health Innovation LTD) and managed using Microsoft Excel. The first author will be responsible for the master copy. Comprehensive Meta-Analysis (version 2, Biostat) software will be used for data synthesis.

### Data collection process

Two investigators (CW and EC) will screen the study titles and abstracts independently to determine eligibility for full-text assessment. The studies must meet the following criteria: (a) adult patients with toxic alcohol ingestion, including co-ingestions; (b) reported intoxication management (including both medical therapies and dialysis) and short-/long-term outcomes; (c) relevant studies of English and French languages will be included. Subsequently, the same investigators will examine the full texts to select those meeting the inclusion and exclusion criteria. Disagreements will be resolved with discussion until consensus is reached or involvement of a third investigator (SH).

### Data extraction

A data extraction form will be created and populated with variables pertaining to the study population and primary and secondary outcomes of interest. The study characteristics of each eligible study including first author, publication year, location of study, study design, number of treatment arms, and follow-up duration will be tabulated. The age, gender, population size, and other baseline demographics such as baseline comorbidities and renal function (if known) will also be recorded. Patient characteristics include intoxicating agent(s), intention of intoxication (e.g., suicide, accidental), serum concentration of toxic alcohol, and time from ingestion/presentation to presentation for medical care. Factors associated with medical interventions include the interval between the time of ingestion to initiation of therapy, administration of antidotes, administration of elimination enhancement therapy (e.g., activated charcoal, gastric lavage), and criteria for proceeding to dialysis (ex. pH). When applicable, the study’s inclusion and exclusion criteria, if available, will also be sought.

In this systematic review, the short- and long-term mortality following toxic alcohol poisoning will be examined. In addition to secondary outcomes outlined above, other short-term secondary outcomes of interest include vasopressor dependence, respiratory failure requiring ventilatory support, renal failure, altered level of consciousness, and duration of hospitalization. Long-term outcomes such as renal recovery, toxin associated complications (for example, visual disturbances due to methanol), vision deficits, other neurologic defects, and cardiovascular events will also be recorded. One reviewer (CW) will collate the above data into Covidence (Veritas Health Innovation LTD) to be reviewed by a second author (EC).

### Quality assessment

The quality of reporting and risk for bias of each included study will be assessed independently by two investigators (CW and EC). Disagreements will be resolved by consensus or involvement of a third reviewer if indicated. Observational studies will be evaluated in accordance with the Cochrane Risk of Bias Assessment Tool for Non-Randomized Studies of Interventions [21], and their quality of reporting will be assessed per Strengthening the Reporting of Observational Studies in Epidemiology (STROBE) Checklist [22]. Qualities of non-randomized trials will be examined per the Risk of Bias In Non-randomized Studies of Interventions (ROBINS-I) tool [23]. The Cochrane Handbook “Risk of Bias” assessment tool will be employed to assess any randomized controlled trials [24].

### Data synthesis

For dichotomous outcome measures, we will calculate a pooled estimate of the proportion of patients across studies and the corresponding 95% confidence interval (CI). For continuous outcomes (e.g., time to dialysis discontinuation), we will calculate a pooled estimate of the mean and a corresponding 95% CI. Pooled analysis will be performed if three or more studies with adequate quality include similar populations, the same type of toxic alcohol intoxication, and outcome(s). The pooled analysis will be performed using the random effects model of DerSimonian and Laird [25]. Study level statistical heterogeneity will be examined utilizing the Cochran’s *Q* and the *I*^2^ test [24]. If high statistical heterogeneity is detected (defined as > 75%), it will be explored using the subgroup analysis specified below. Publication bias will be assessed by visual examination of the funnel plot and using Egger’s test [26]. Pre-specified subgroup analyses will be performed based on the type of toxic alcohol poisoning, dialysis use, and medical intervention received. Type of alcohol poisoning will be assessed as a separate subgroup given that different types of alcohol poisoning have different pathophysiologic mechanisms and can be expected to have different outcomes [1]. In addition, the use of dialysis and other medical interventions (e.g., fomepizole treatment) is often employed in more severe cases in which worse outcomes might be expected than the overall patient population with toxic alcohol poisoning [1]. If sufficient intervention trials pertaining to the review topic are found and included, then an examination of the quality of evidence will be performed using the Grading of Recommendations Assessment, Development and Evaluation (GRADE) criteria [27].

### Potential limitations

We anticipate potential limitations to our review due to the likelihood that there will be a wide heterogeneity across the included studies with respect to the types of toxic alcohol poisoning, differences in reported outcomes, absence of comparator groups in many studies, and differences in patients’ characteristics at baseline. At the review level, we expect challenges with respect to the possibility that many studies will be case series in which case the selection of patients for inclusion is less likely to be representative of the overall population of patients with toxic alcohol poisoning.

### Protocol amendments

Any protocol amendments will be clearly documented and justified with an addendum made to the protocol specifying the changes and their justification. In addition, any such changes and their justification will be included in the final report of the review.

## Discussion

Toxic alcohols are readily accessible in household and hardware stores [1]. Accidental and intentional poisoning through ingestion remains prevalent [3]. Understanding the mechanisms by which each alcohol imparts its toxicologic effects has aided in the development of antidotes and the role for renal replacement therapy (in particular, hemodialysis). However, there is likely variation in treatment practice due to the lack of robust consensus regarding thresholds for initiating medical therapies such as antidotes and renal replacement therapy [1, 2, 4]. In addition, little is known about long-term outcomes. This systematic review will examine the short- and long-term outcomes of patients after toxic alcohol poisoning and with respect to the treatments they did or did not receive. In doing so, we hope it will help shed light on the impact of various treatments on outcomes and help inform the optimal management and follow-up after toxic alcohol poisonings.

### Additional file

Additional file 1: PRISMA-P 2015 Checklist (DOCX 35 kb)

## Appendix

### MEDLINE search strategy

1. exp. Renal Replacement Therapy/
2. ((kidney* or renal) adj3 replacement adj2 therap*).tw.
3. exp. Renal Dialysis/
4. ((renal or extracorporeal) adj2 dialys#s).tw.
5. exp. Hemofiltration/
6. h?mofiltration*.tw.
7. h?modialys#s.tw.
8. h?modialfiltration*.tw.
9. Dialysis/
10. cavf.tw.
11. sustained low-efficiency dialysis.tw.
12. sled.tw.
13. or/1–12
14. ethylene glycols/ or chloral hydrate/ or chloralose/ or ethylene glycol/ or methoxyhydroxyphenylglycol/ or polyethylene glycols/ or cetomacrogol/ or hydrogel, polyethylene glycol dimethacrylate/ or nonoxynol/ or octoxynol/ or poloxalene/ or poloxamer/ or polysorbates/
15. (toxic adj2 alcohol*).tw.
16. (ethylene adj2 glycol*).tw.
17. ethanediol*.tw.
18. hydroxyethanol*.tw.
19. (monoethylene adj2 glycol*).tw.
20. (diethylene adj2 glycol*).tw.
21. polyhydroxyethyl*.tw.
22. hydrogel*.tw.
23. (advantage adj S).tw.
24. (delfen adj cream*).tw.
25. (emulgen adj “911”).tw.
26. (emulgin adj “913”).tw.
27. methanol*.tw.
28. (chloral adj hydrate*).tw.
29. dihydroxyethane*.tw.
30. noctec.tw.
31. chloralose.tw.
32. anhydroglucochloral*.tw.
33. glucochloral*.tw.
34. cetomacrogol*.tw.
35. nonoxynol*.tw.
36. octoxynol*.tw.
37. poloxalene*.tw.
38. poloxamer*.tw.
39. polysorbate*.tw.
40. methoxyhydroxyphenylglycol*.tw.
41. methoxyphenylethyleneglycol*.tw.
42. (methoxyphenylethylene adj glycol*).tw.
43. mopeg.tw.
44. mhpg.tw.
45. vanylglycol*.tw.
46. (polyethylene adj2 (oxide* or glycol*)).tw.
47. macrogol*.tw.
48. polyoxyethylene*.tw.
49. triton*.tw.
50. polyethyleneoxide*.tw.
51. nonoxinol*.tw.
52. (patentex adj oral).tw.
53. pluronic*.tw.
54. proxanol*.tw.
55. octoxinol*.tw.
56. or/14–55
57. exp. Adult/ or (adult or adults or adulthood or middle age or middle aged or elderly or senior or seniors or man or men or woman or women).tw.
58. 13 and 56 and 57

## Abbreviations

EMBASE: Excerpta Medica dataBASE
ECRT: Extracorporeal renal replacement therapy
EXTRIP: Extracorporeal Treatments in Poisoning
GRADE: Grading of Recommendations Assessment, Development and Evaluation
MEDLINE: Medical Literature Analysis and Retrieval System Online
PRISMA-P: Preferred Reporting Items for Systematic Review and Meta-Analysis Protocols
PROSPERO: International Prospective Register of Systematic Reviews
ROBINS-I: Risk of Bias In Non-randomized Studies of Interventions
STROBE: Strengthening the Reporting of Observational Studies in Epidemiology
